# METTL3 promotes cell cycle progression via m^6^A/YTHDF1-dependent regulation of *CDC25B* translation

**DOI:** 10.7150/ijbs.70335

**Published:** 2022-05-01

**Authors:** Huifeng Li, Ying Zhong, Guangxu Cao, Hezhan Shi, Yiyao Liu, Lingfeng Li, Peidi Yin, Jialing Chen, Zhendong Xiao, Bin Du

**Affiliations:** 1 Department of Pathology, Shanghai First Maternity and Infant Hospital, Tongji University, Shanghai 200120, China.; 2 Department of Pathology, School of Medicine, Jinan University, Guangzhou 510632, China.; 3 Department of Gynecology, Shanghai East Hospital, School of Medicine, Tongji University, Shanghai 200120, China.; 4 The Third Affiliated Hospital, Sun Yat-Sen University, Guangzhou 510630, China.

**Keywords:** METTL3, cell cycle, m^6^A

## Abstract

The cell cycle machinery controls cell proliferation and the dysregulation of the cell cycle lies at the heart of carcinogenesis. Thus, exploring the unknown regulators involved in the cell cycle not only contribute to better understanding of cell proliferation but also provide substantial improvement to cancer therapy. In this study, we identified that the expression of methyltransferase METTL3 was upregulated in the M phase. Overexpression of METTL3 facilitated cell cycle progression, induced cell proliferation *in vitro* and enhanced tumorigenicity *in vivo*, while knockdown of METTL3 reversed these processes. METTL3 induced* CDC25B* mRNA m^6^A modification in the M phase, which accelerated the translation of *CDC25B* mRNA through YTHDF1-dependent m^6^A modification. Clinical data analysis showed that METTL3 and CDC25B were highly expressed in cervical cancer. Our work reveals that a new mechanism regulates cell cycle progression through the METTL3/m^6^A/CDC25B pathway, which provides insight into the critical roles of m^6^A methylation in the cell cycle.

## Introduction

As a ubiquitous and complex process, the cell cycle is the core event that regulates cell proliferation, and it is closely related to carcinogenesis 1, 2. The cell cycle includes DNA synthesis (S) and mitosis (M) phases, which are separated by a gap phase; the phases progress in the order G1-S-G2-M. Cell cycle transitions are mainly driven by Cyclins and Cyclin-Dependent Kinases (CDKs) 3. Moreover, there are a series of checkpoints to ensure that each stage of the cell cycle is completed before the next stage is initiated. Progression through the G1 phase is mainly controlled by Cyclin D1 and CDK4/6, while the CDK2/Cyclin E complex controls progression from the G1 phase to the S phase. The G2/M transition is driven by CDK1/Cyclin B complexes and many other regulators, such as WEE1(WEE1 G2 checkpoint kinase), PLK1(polo like kinase 1) and cell division cyclin 25 (CDC25) 4. The CDC25 family includes three homologous isomers (CDC25A, CDC25B and CDC25C) and is involved in cell cycle progression by controlling the phosphorylation state of CDKs. CDC25B, considered a “trigger” phosphatase, is associated with the initial activation of CDK1/cyclin B at the centrosome during the G2/M transition 5, 6. Additionally, CDC25B is reported to be a potential oncogene that is highly expressed in tumors 7-9. Previous studies show that cell cycle dysregulation is a central hallmark of oncogenesis and that the activity of cellular proteins implicated in cell cycle regulation is frequently altered in tumor cells, which protects tumor cells from different types of stress and promotes tumor progression 9, 10. Thus, cell cycle regulators are expected to be promising targets for cancer therapy. Palbociclib (CDK4/6 inhibitor) and Milciclib (pan-CDK inhibitor) have been approved by the Food and Drug Administration (FDA), representing a promising new area of anticancer drug development 11, 12. However, series of unknown regulators involves in the cell cycle remaining to be researched.

N6-Methyladenosine (m^6^A) is the most common internal modification of eukaryotic mRNAs 13. This reversible and dynamic process is mainly catalyzed by methyltransferase complexes, including methyltransferase-like 3 (METTL3)/methyltransferase like 14 (METTL14) and Wilms' tumor 1-associating protein (WTAP), which have been designated “writers”. m^6^A modification can be reversed by m^6^A demethylases (erasers), such as fat mass and obesity-associated protein (FTO) and AlkB homolog 5 (ALKBH5). Factors that recognize and bind to specific modifications have been identified as “readers” and mainly include the YTH m^6^A RNA binding protein 1-3 (YTHDF1-3), YTH domain-containing 1-2 (YTHDC1-2) and insulin-like growth factor 2 mRNA binding protein 2 (IGF2BP2) 14-18. Accumulating evidence has shown that m^6^A modifications can regulate mRNA fate and function by influencing mRNA stability, splicing, transport, localization and translation. Moreover, m^6^A modifications participate in many diverse biological processes, including tissue development, DNA damage responses, sex determination, and tumorigenesis 19-21.

m^6^A modification has been implicated in regulating the expression of genes related to the cell cycle. It was reported that FTO regulates cell cycle progression in an m^6^A-CCNA2-YTHDF2-dependent manner 22. Downregulation of YTHDF1 expression inhibits non-small-cell lung carcinoma (NSCLC) cell proliferation by regulating the translation of *CDK2*, *CDK4*, and *CCND1* 23. IGF2BP3, one of the m^6^A readers, regulates the cell cycle progression and angiogenesis of colon cancer by reading the m^6^A modifications of *CCND1* and *VEGF,* respectively 24. These studies suggest that m^6^A modification potentially plays a role in cell cycle regulation. However, how transcriptomic m^6^A changes in every phase of the cell cycle, including the G1, S, G2, and M phases, and its function remain unexplored.

In the current study, we found that METTL3 expression was upregulated and was associated with high m^6^A levels in the M phase of the cell cycle. Functionally, METTL3 facilitated cell cycle progression and induced cell proliferation *in vitro* and *in vivo*. In addition, we identified *CDC25B* as the downstream target of METTL3 and verified that the METTL3-modulated m^6^A modification of *CDC25B* was recognized by YTHDF1, contributing to the translation of *CDC25B* mRNA in the M phase. Overall, our work is the first to reveal that METTL3 regulates cell cycle progression by controlling the G2/M transition in an METTL3-m^6^A-CDC25B-dependent manner, which provides insight into the critical roles of m^6^A methylation in cell cycle regulation.

## Results

### METTL3 expression was elevated and correlated with the highest m^6^A level in the M phase

**Figure 1:**
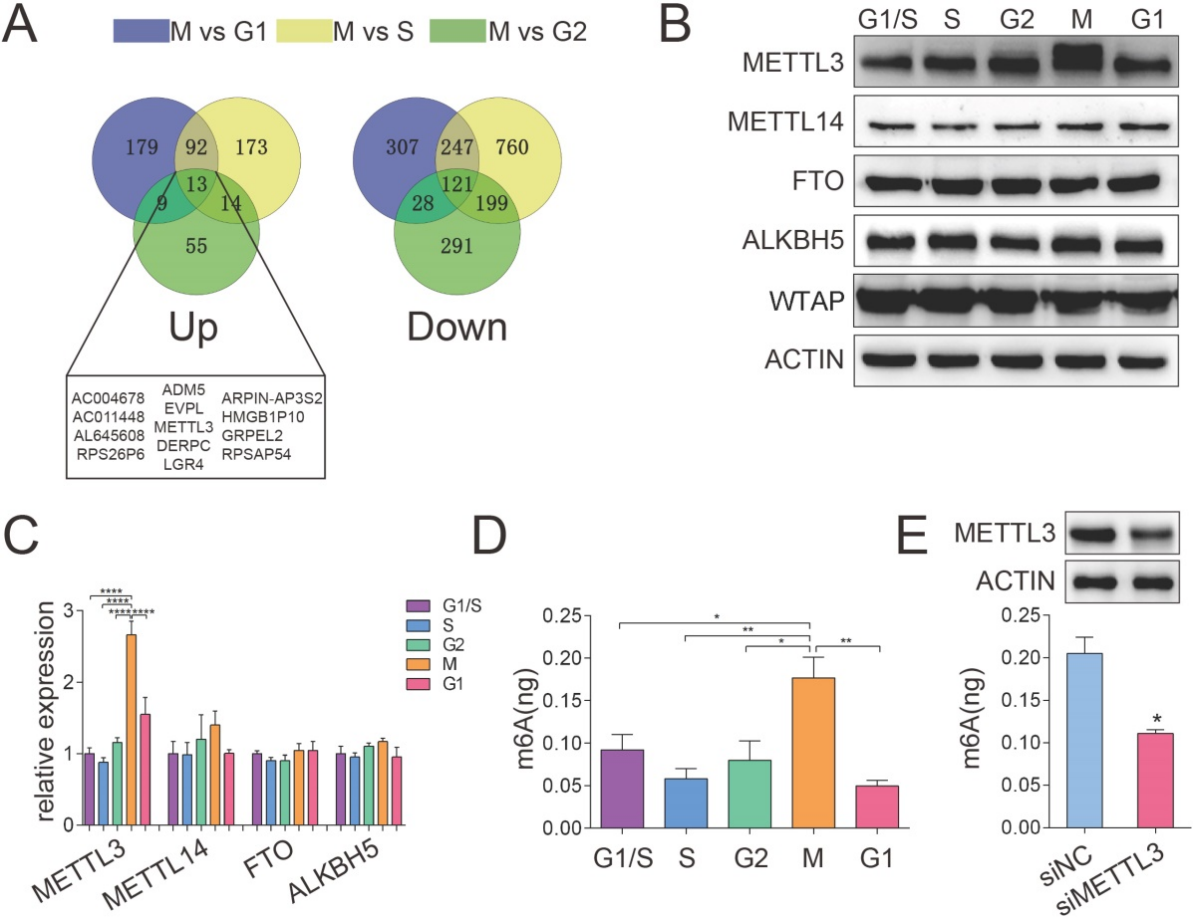
** METTL3 expression was elevated and correlated with high m^6^A levels in the M phase.** (A) Venn diagram illustrated the upregulated and downregulated genes differentially expressed in the M phase compared to other cell cycle phases (G1 phase, S phase and G2 phase). (B) The protein levels of METTL3, METTL14, FTO, ALKBH5 and WTAP were analyzed by Western blotting. ACTIN served as a loading control. (C) The relative mRNA levels of METTL3, METTL14, FTO and ALKBH5 were analyzed by qPCR. **** P < 0.0001 (one-way ANOVA analysis) for the indicated comparisons. Relative mRNA expression levels were normalized by 18S rRNA. Data are presented as the mean ± SD for n = 3 replicate cultures. (D) Global m^6^A mRNA methylation levels in various HeLa cell cycle phases were determined by m^6^A enzyme-linked immunosorbent assays. m^6^A RNA methylation quantification was performed based on the standard curve. * P < 0.05, ** P < 0.01 (one-way ANOVA analysis) for the indicated comparisons. (E) Quantification of m^6^A enrichment in mRNA from control and siMETTL3 cells in the M phase. The knockdown efficiency of METTL3 in HeLa cells was confirmed by Western blotting indicated in the upper panel. Quantification of m^6^A was detected by the m^6^A RNA methylation quantification kit. Data shown are mean ± SD for n = 3 replicate cultures, * P < 0.05 compared to negative control. (The western blotting data are representative of n=3 independent experiments).

To obtain the transcriptional profiles of the HeLa cell cycle, we performed cell cycle synchronization by using the double-block method with thymidine and nocodazole. The method described in Figure S1A utilizes a double thymidine block (an inhibitor of DNA synthesis) followed by treatment of cells with nocodazole (a mitotic inhibitor) to obtain large cell populations at distinct phases of the cell cycle. The cell cycle synchronization efficiency was confirmed by Western blotting and qPCR analyses of markers including CCNE, CCNA2 and CCNB1, which represent the S, G2 and M phases, respectively. The results shown in Figure S1B and Figure S1C indicated that the cell cycle synchronization model was successfully established, which was consistent with a previous study 25. According to DESeq analysis (based on the following criteria: fold change > 2 and P < 0.05), we identified differentially expressed genes of each comparison group. As a process when one parent cell physically divides into two daughter cells, the M phase might be the most active phase of the mitosis cell cycle. Comprehensive and systematic investigations of the mechanisms regulating the M phase may provide important clues to gain an extensive understanding of cell cycle regulation. Thus, we analyzed the differentially expressed genes in M phase compared to other cell cycle phases (G1 phase, S phase and G2 phase). Totally, 13 up-regulated genes and 121 down-regulated genes were identified (Figure 1A). Interestingly, METTL3, one of the main methyltransferase, was upregulated in the M phase compared to other phases, which were further confirmed by qPCR and Western blotting assays (Figure 1B). Moreover, we compared the m^6^A distribution in each phase of the cell cycle and found increased global m^6^A levels in the M phase (Figure 1D). Furthermore, the mRNA and protein expression levels of the primary m^6^A methyltransferase and demethylase genes in the G1, S, G2 and M phases were also detected by qPCR and Western blotting assays. In addition to METTL3, there was no significant difference in the expression of the other main m^6^A methyltransferase and demethylase genes during cell cycle progression (Figure 1B and 1C). Moreover, the knockdown of METTL3 significantly downregulated the m^6^A level in cells in the M phase (Figure 1E), which confirmed the methylation activity of METTL3 in the cell cycle. Overall, our results indicated that METTL3 expression is elevated and correlated with the highest m^6^A level in the M phase. Recently, METTL3 has been reported to regulate the S-phase progression in the cell cycle of fibroblasts through the METTL3-YTHDF2-CCND1 pathway, which ultimately affects fat formation 26. This result implied that METTL3 and even m^6^A modification may be associated with different regulatory pathways in various types of cells and different pathological processes.

### METTL3 significantly facilitated cell cycle progression and promoted cell growth *in vitro*

**Figure 2:**
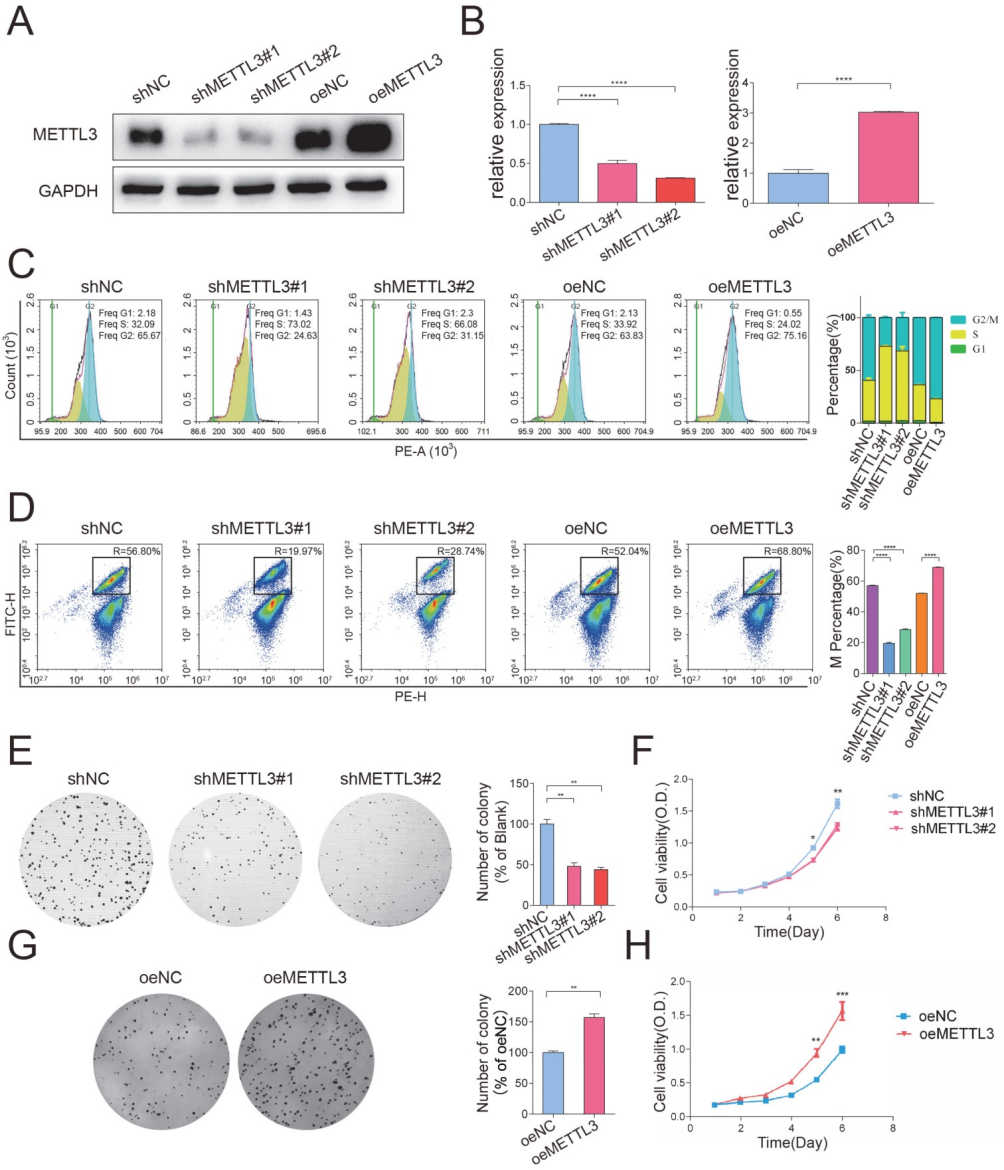
** METTL3 facilitated cell cycle progression and promoted cell growth *in vitro*.** (A) The knockdown and overexpression efficiency of METTL3 in HeLa cells was confirmed by Western blot. Western blot analysis of protein extracted from METTL3 stable knockdown and overexpression HeLa cells. The western blotting data are representative of n=3 independent experiments. (B) The knockdown and overexpression efficiency of METTL3 in HeLa cells was also confirmed by RT-qPCR analysis. Gene expression was assayed by RT-qPCR analysis of RNA extracted from METTL3 stable knockdown and overexpression HeLa cells. Data shown are mean fold-change values ± SD for n = 3 replicate cultures, with intra-sample normalization to the 18S RNA level of each sample. **** P < 0.0001 for the indicated comparisons. (C) Cell cycle progression after knockdown (shNC or shMETTL3) or overexpression (oeNC or oeMETTL3) of METTL3 was detected by flow cytometry. The percentage of cells in the G1, S and G2/M phases were calculated using FACS software and data were shown in each image on the left panel. The average percentage of cells in the G1, S and G2/M phases from 3 independent experiments were shown by a stacked bar chart on the right panel. (D) The percentage of cells in the M phase was detected by phospho-H3-specific flow cytometry in METTL3-knockdown cells, METTL3-overexpression cells, and the corresponding negative control cells. The percentage of cells in the M phases showed in each image on the left panel. The average percentage of cells in the M phases from 3 independent experiments was showed by stacked bar chart on the right panel. Data shown are mean ± SD for n = 3 replicate cultures. **** P < 0.0001 (one-way ANOVA analysis) for the indicated comparisons. (E-F) Colony formation assays (E) and CCK-8 proliferation assays (F) were performed to evaluate the proliferation of HeLa cells after transfection of shMETTL3 interference plasmid. Histograms present the colony numbers of each group. Data shown on (E) the right panel are mean fold-change values ± SD for n = 3 replicate cultures. * P < 0.05 (one-way ANOVA analysis) for the indicated comparisons. (F) Data shown are mean ± SD for n = 3 replicate cultures. * P < 0.05, ** P < 0.01 for METTL3 knockdown versus control, evaluated on indicated day (one-tailed t-test). (G-H) Colony formation assays (G) and CCK-8 proliferation assays (H) were performed to evaluate the proliferation of HeLa cells after transfection of METTL3 overexpression plasmid. Histograms present the colony numbers of each group. Data shown at (G) right panel are mean fold-change values ± SD for n = 3 replicate cultures. ** P < 0.01 (unpaired Student's t-test) for the indicated comparisons. (H) Data shown are mean ± SD for n = 3 replicate cultures. ** P < 0.01, *** P < 0.001 for METTL3 overexpression versus control, evaluated on indicated day (one-tailed t-test).

To determine the biological function of METTL3, we established stable METTL3-knockdown and METTL3-overexpression HeLa cells. The transfection efficiency was confirmed by Western blotting (Figure 2A) and qPCR (Figure 2B). To investigate the effect of METTL3 on cell cycle progression, METTL3-knockdown and METTL3-overexpression cells were harvested 10 hours after nocodazole was added, and then, flow cytometry assays were performed. The percentage of cells in each phase of the cell cycle showed that METTL3 knockdown delayed cell cycle progression, while METTL3 overexpression significantly facilitated cell cycle progression (Figure 2C). We further carried out an H3-specific flow cytometry assay to detect whether METTL3 is necessary for the G2/M transition. As shown in Figure 2D, the knockdown of METTL3 significantly decreased the percentage of cells in the M phase, whereas overexpression of METTL3 elevated the percentage of cells in the M phase. Furthermore, colony formation and CCK-8 assays were used to detect the effect of METTL3 on cell proliferation. The results of these experiments showed that the growth and proliferation capabilities of the cells were weakened after the knockdown of METTL3 (Figure 2E and Figure 2F), while the overexpression of METTL3 exerted the opposite effect (Figure 2G and Figure 2H). Collectively, these data suggested that METTL3 significantly facilitated cell cycle progression and promoted cell growth *in vitro*.

### Transcriptome-wide m^6^A-seq identified potential targets of METTL3

**Figure 3:**
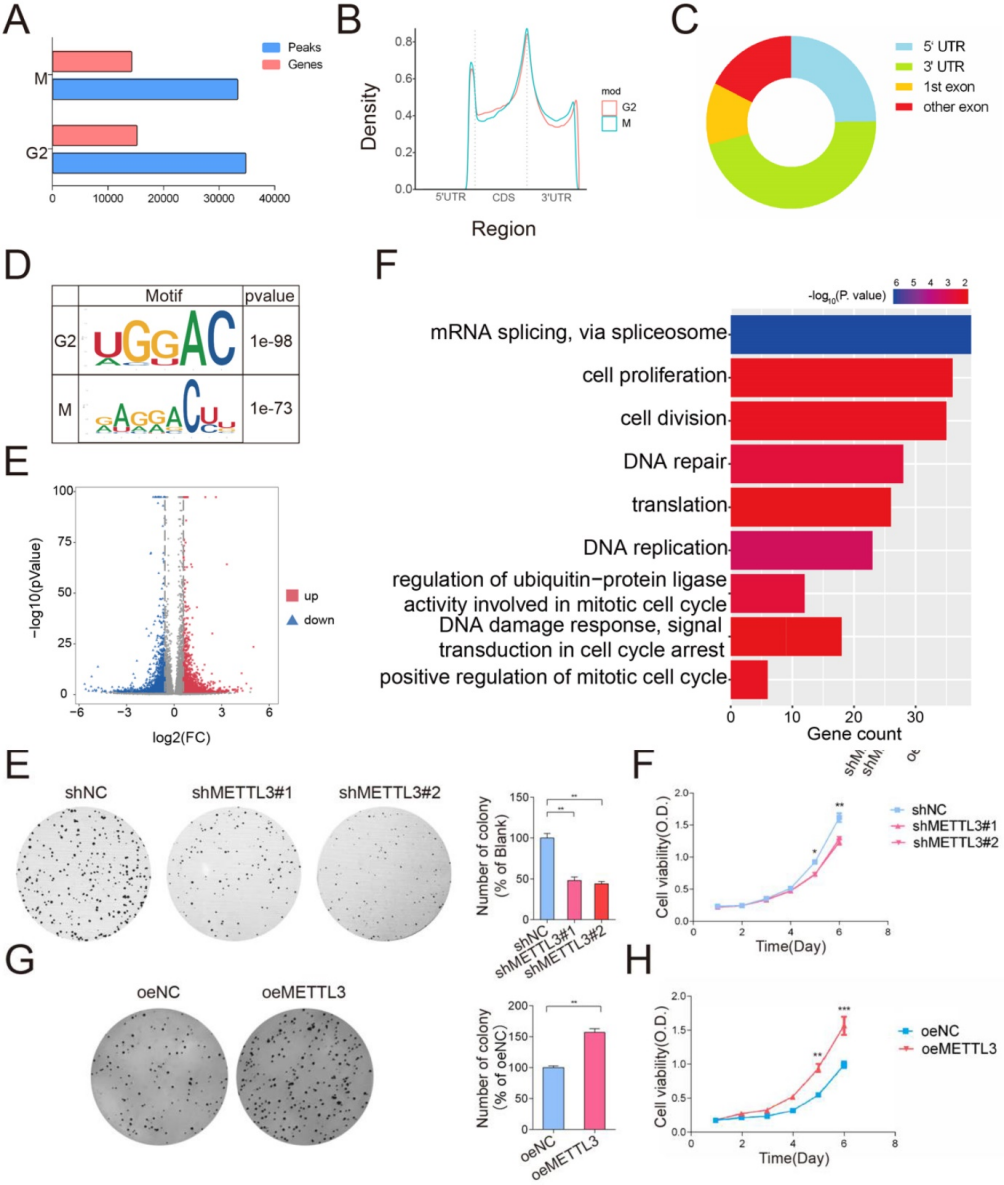
** m^6^A -seq analysis of cells in the M and G2 phases.** (A)The eluted RNA and MeRIPed RNA from HeLa cells at M or G2 phases were analyzed by deep sequencing. Bar plot showing the numbers of m^6^A peaks and m^6^A -modified genes in the M and G2 phases. (B) Density distribution of the m^6^A peak across mRNA transcripts. m^6^A peak distribution in the 5′UTR, start codon, CDS, stop codon or 3′UTR region across the entire set of mRNA transcripts**.** (C) Regions of the 5′ untranslated region (5′UTR), coding region (CDS), and 3′untranslated region (3′UTR) were split into 100 segments, then percentages of m^6^A peaks that fall within each segment were determined. Pie chart showing the different m^6^A peak distribution between the 5'UTR, exon and 3'UTR regions. (D) Predominant consensus motif GGAC was detected in both G2 phase and M phase in m^6^A-seq. (E) Volcano plot showing the distribution of genes with differential m^6^A levels in the M phase groups compared with the G2 phase group. (F) Gene ontology (GO) analysis of genes with increased m^6^A methylation levels (Fold Change > 2; P < 0.05 in m^6^A-seq) in the M phase compared to the G2 phase. Gene counts of GO terms were exhibited on the x-axis and p-values were indicated with the color of columns.

Considering that METTL3 expression was upregulated in the M phase and considering the critical role of the G2/M transition in the cell cycle 27, we used m^6^A-seq analysis of the G2 phase and M phase to investigate the potential molecular mechanism of METTL3 and to identify its downstream targets in the cell cycle. m^6^A -seq identified 34644 peaks in 15,097 genes in the G2 phase (peaks and genes identified in the G2 phase are listed in Table S3) and 33228 peaks in 14,109 genes in the M phase (peaks and genes identified in the M phase are listed in Table S4). Among these genes, 12,514 genes were common between the G2 and M phases (Figure 3A). The genomic distribution of m^6^A peaks across mRNA transcripts and the m^6^A motifs identified from the two groups (Figure 3B-3D, mapped region of each sample are shown in Figure S1D) were consistent with those reported previously 28. Moreover, the m^6^A -seq analysis revealed that the m^6^A peaks of 1,895 transcripts were increased in the M phase compared with the G2 phase (Figure 3E and transcripts list in Table S5) (fold change > 1.5, P < 0.05) (Different m^6^A peaks and Transcription Start Sites are shown in Figure S1E, the density of Different Expression Genes is shown in Figure S1F). To obtain comprehensive gene function information, Gene Ontology (GO) analysis was conducted. The analysis revealed that the differentially m^6^A -modified genes were significantly enriched in gene sets involved in cell cycle regulation, such as “DNA damage response, signal transduction in cell cycle regulation” (P < 0.01, 0.4% of cluster frequency) and “positive regulation of mitotic cell cycle” (P < 0.01, 0.02% of cluster frequency). In addition, the m^6^A-modified genes were also involved in regulating mRNA splicing and translation, which are directly related to the functional consequence of m^6^A modification (Figure 3F and GO_BP list in Table S6).

### m^6^A methylation regulated CDC25B activation

**Figure 4:**
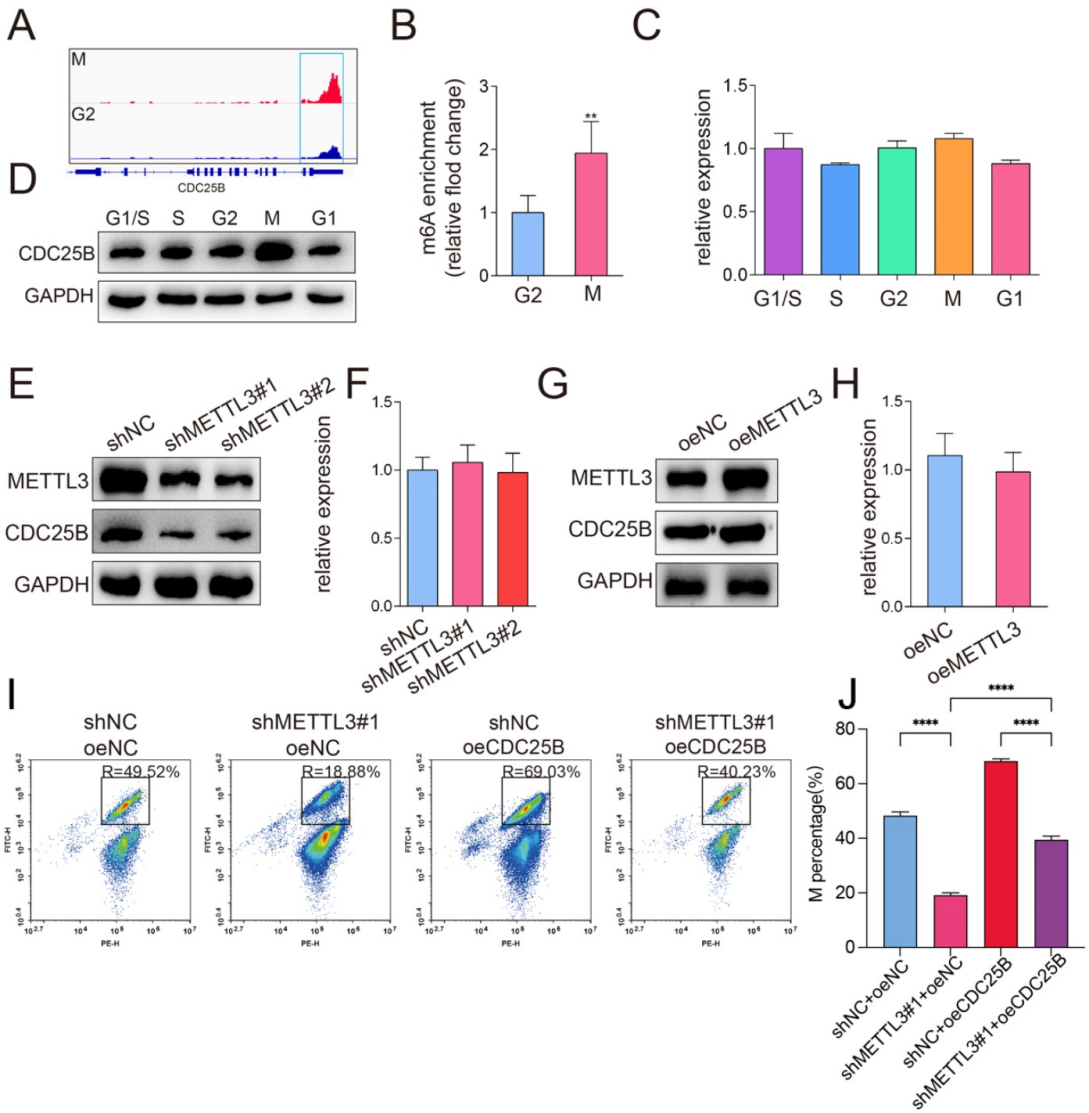
** CDC25B was a downstream target of METTL3-mediated m^6^A modification.** (A) The m^6^A abundances on* CDC25B* mRNA in the G2 phase and the M phase were plotted by IGV (Integrative Genomic Viewer). *CDC25B* mRNA had visible abundant modifications of m^6^A in the 3' UTR region in M phase cells compared to G2 phase cells. (B) The m^6^A levels of *CDC25B* in the G2 phase and the M phase were quantified by m^6^A RNA immunoprecipitation followed by RT-qPCR. The relative m^6^A level was normalized by the input. Data shown are mean fold-change values ± SD of triplicate experiments, ** P < 0.01 (unpaired Student's t-test) M verse G1 phase. (C-D) The mRNA (C) and protein (D)expression of CDC25B in various phases of the cell cycle was analyzed by RT-qPCR or Western blotting. (C) There is no significance of *CDC25B* mRNA between various phases of the cell cycle. (E) Western blotting of METTL3 and CDC25B in HeLa cells when METTL3 was knocked down. GAPDH served as a loading control. (F) qPCR analysis of *CDC25B* mRNA in HeLa cells when METTL3 was knocked down. There is no significant between the 3 groups. (G) Western blotting of METTL3 and CDC25B in HeLa cells when METTL3 was overexpressed. GAPDH served as a loading control. (H) qPCR analysis of *CDC25B* mRNA in HeLa cells when METTL3 was overexpressed. There is no significance between the two groups. (The western blotting data are representative of n=3 independent experiments). (I) The percentage of cells in the M phase was detected by phospho-H3-specific flow cytometry in HeLa cells. The percentage of cells in the M phases showed in each image. (J) The average of the percentage of cells in the M phases from 3 independent experiments was shown by stacked bar chart on the right panel. Data shown are mean ± SD for n = 3 replicate cultures. **** P < 0.0001 (one-way ANOVA analysis) for the indicated comparisons.

We next screened the genes listed in the cell cycle-related GO category in our data. Six genes (*SHB*, *EIF4EBP1*, *PKN2*, *PAFAH1B1*, *CDC25B*, *TERF1*) were involved in the “positive regulation of mitotic cell cycle” term. Among these genes, *CDC25B* was identified as a direct target of m^6^A modification in our in-house m^6^A -seq data and RMBase, which integrated 477,452 m^6^A modifications from 566 datasets 29. The m^6^A peak detected near the 3'UTR of *CDC25B* was dramatically increased in the M phase compared with the G2 phase (Figure 4A). Previous studies also have shown that CDC25B plays a critical role in the G2-to-M phase transition 30, as well as cancer progression 31. In light of such a critical role CDC25B played in cell cycle and carcinogenesis, we selected CDC25B for further investigation. To determine whether *CDC25B* is a substrate of METTL3, we validated our m^6^A -seq data by m^6^A RIP-qPCR assay. The results confirmed that the m^6^A level of *CDC25B* was markedly increased in the M phase compared with the G2 phase (Figure 4B) (P < 0.01). Moreover, the protein expression of CDC25B was upregulated in the M phase (Figure 4D). In contrast, the mRNA levels of *CDC25B* remained constant throughout the cell cycle (Figure 4C). Next, we investigated the regulation of CDC25B expression by METTL3 through Western blotting and qPCR analyses. Western blotting analysis showed that CDC25B expression was decreased when METTL3 knocked down (Figure 4E) and was significantly increased when METTL3 overexpressed (Figure 4G). However, overexpression or knockdown of METTL3 did not obviously affect the *CDC25B* mRNA levels (Figure 4F and 4H). Further, H3-specific flow cytometric analysis showed that overexpression of CDC25B was able to restore the proportion of cells in the M phase that had been significantly reduced upon knockdown of METTL3 (Figure 4I-J). Taken together, these findings proved that *CDC25B* is a downstream target of METTL3-mediated m^6^A modification and suggested that the regulation of CDC25B by METTL3 might affect translation.

### YTHDF1 enhanced *CDC25B* mRNA translation in an m^6^A-dependent manner

**Figure 5:**
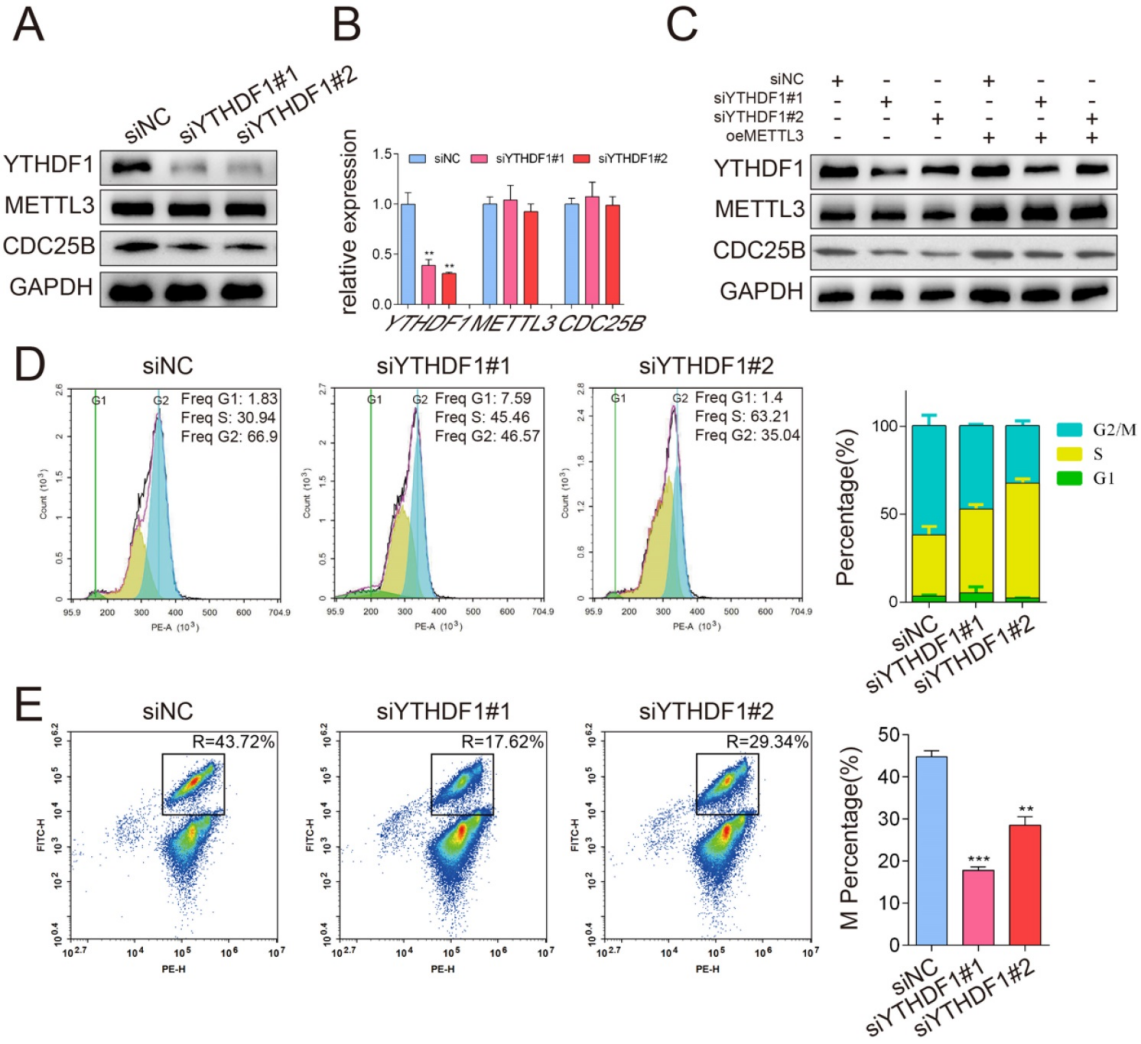
** YTHDF1 enhanced *CDC25B* mRNA translation in an m^6^A -dependent manner.** (A) Western blotting of METTL3 and CDC25B after YTHDF1 inhibition in HeLa cells. GAPDH served as a loading control. (B) RT-qPCR analysis of *METTL3* and *CDC25B* mRNA expression after YTHDF1 inhibition in HeLa cells. Data shown are mean fold-change values ± SD for n = 3 replicate cultures. ** P < 0.01 (one-way ANOVA analysis) compared to the negative control (siNC) group. (C) Analysis of the CDC25B protein expression in HeLa cells co-transfected with YTHDF1 siRNA and METTL3 plasmid. GAPDH served as a loading control. (D) Changes in the cell cycle after knockdown of YTHDF1 were detected with flow cytometry. The percentage of cells in the G1, S and G2/M phases were calculated using FACS software and data were shown in each image on the left panel. The average percentage of cells in the G1, S and G2/M phases from 3 independent experiments was shown by a stacked bar chart at the right panel. (E) The percentage of cells in the M phase after knockdown of YTHDF1 was detected by phospho-H3-specific flow cytometry. The percentage of cells in the M phases showed in each image on the left panel. The average percentage of cells in the M phases from 3 independent experiments was shown by a stacked bar chart on the right panel. Data shown are mean values ± SD for n = 3 replicate cultures. ** P < 0.01, *** P < 0.001 (one-way ANOVA analysis) compared to the negative control (siNC) group. (The western blotting data are representative of n=3 independent experiments).

Previous studies have shown that the m^6^A “reader” protein YTHDF1 specifically recognizes m^6^A modification and mediates m^6^A -containing mRNA translation 19, which led us to hypothesize that the translation levels of m^6^A -modified *CDC25B* mRNA might be regulated by YTHDF1. To test our hypothesis, we conducted a loss-of-function assay by using two small interfering RNAs (siRNAs) that targeted YTHDF1. The protein and mRNA expression levels were then examined by Western blotting and qPCR. Our analysis revealed that the protein expression of CDC25B was markedly suppressed in YTHDF1-depleted cells (Figure 5A), whereas the relative mRNA expression of *CDC25B* was not significantly different (Figure 5B). To further determine whether METTL3 enhanced *CDC25B* mRNA translation through YTHDF1, rescue experiments were carried out. We first overexpressed METTL3 in HeLa cells and then downregulated YTHDF1 by using siRNA. The results showed that the downregulation of YTHDF1 could weaken the upregulation of CDC25B via METTL3 overexpression (Figure 5C). Functionally, consistent with the results of METTL3 knockdown, loss of YTHDF1 also delayed cell cycle progression (Figure 5D) and decreased the percentage of cells in the M phase (Figure 5E). Together, these results demonstrate that METTL3 and YTHDF1 cooperatively mediate the translational activity of *CDC25B* mRNA and further influence cell cycle progression.

### METTL3 promoted tumor growth *in vivo*

**Figure 6:**
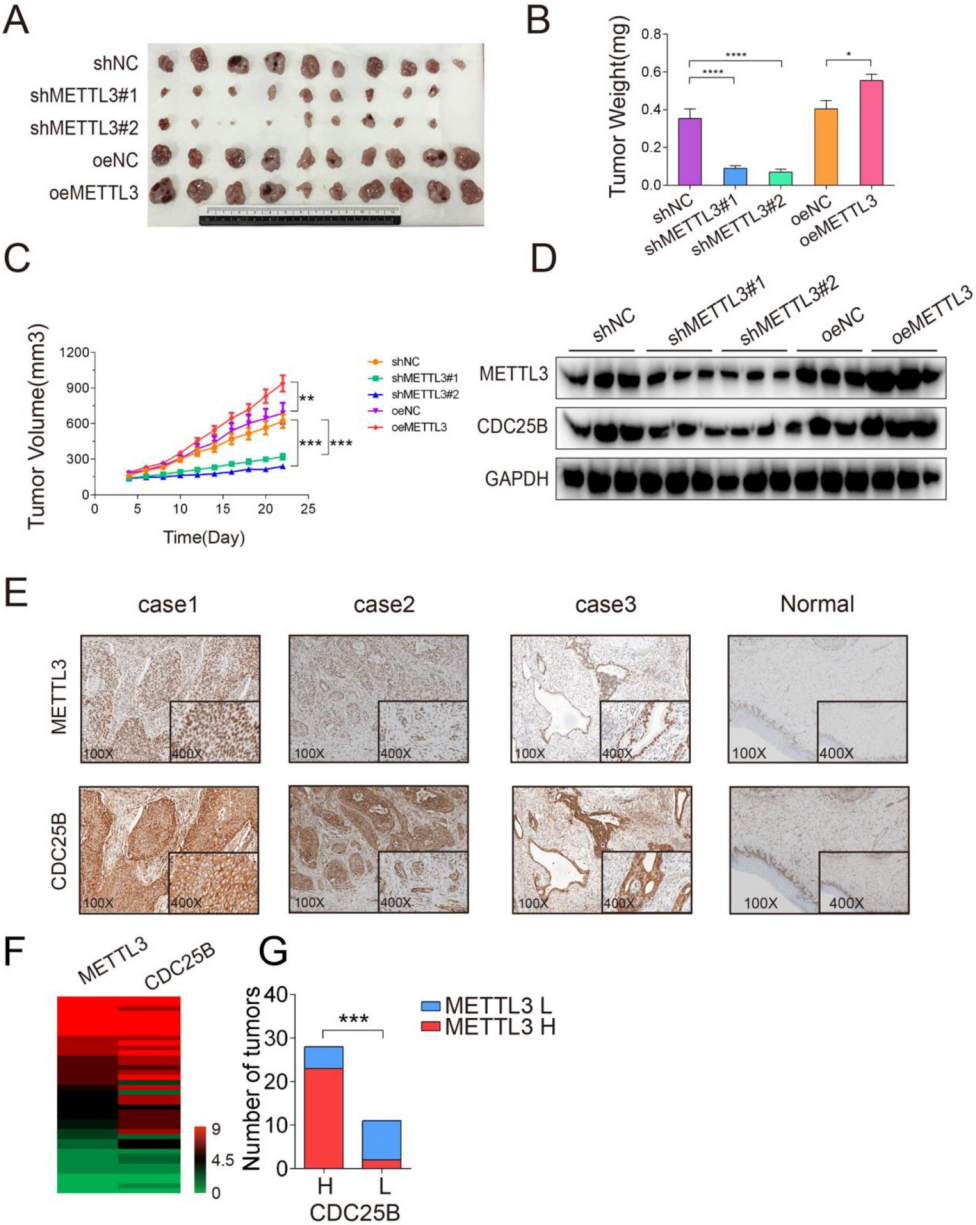
** METTL3 promoted tumor growth *in vivo*.** (A) Representative images of subcutaneous HeLa tumors growth in xenografted BALB/c nude mice. Each group of mice was ectopically implanted with 5 × 10^6^ indicated cells into the flanks of mice (n > 9). Here, cells were transfected with shRNA (shNC or shMETTL3) or overexpression (oeNC or oeMETTL3) lentiviral vector. (B) Tumors were harvested 22 days after cell injection, and the weights of the tumors that formed in the subcutaneously implanted mouse model were measured. The data are presented as the mean ± SEM tumor weight, * P < 0.05, **** P < 0.0001(one-way ANOVA analysis) for the indicated comparisons. (C) The size of the tumors that formed in the subcutaneously implanted mouse model was monitored every 2 days. The data are presented as the mean ± SEM tumor volume, ** P < 0.01, *** P < 0.001(one-tailed t-test) for the indicated comparisons. (D) Western blotting assay of METTL3 and CDC25B protein expression in the tumors from each group. Data are representative of 3 different tumors from each group. GAPDH served as a loading control. (E) Immunohistochemical staining of the METTL3 and CDC25B protein in human cervical tumors (n=39). (Magnification: 100× and 400×). IHC images of patients 1 to 3 were selected to show collaborative high expression of METTL3 and CDC25B, whereas images of patient 4 displayed low expression of these proteins. (F) Heatmap displayed the IHC scores of METTL3 and CDC25B in 39 human cervical tumor samples. (G) The Number of tumors expressing high (H) or low (L) levels of CDC25B in tumors with high (H) or low (L) METTL3 expression. *** P < 0.001 (Student's t-test)

To test the effect of METTL3 on tumorigenicity, we performed a subcutaneous implantation experiment in nude mice. Stable METTL3-knockdown, METTL3-overexpression and control HeLa cells were established and subcutaneously injected into the flanks of nude mice (Figure 6A). Knockdown of METTL3 dramatically suppressed HeLa subcutaneous tumor growth in nude mice, as reflected by the significant reduction in tumor size and tumor weight, whereas METTL3 overexpression resulted in increased tumor size and tumor weight (Figure 6B-6C). In addition, consistent with the results of the *in vitro* experiment, the expression of CDC25B was decreased in METTL3-knockdown tumors but significantly increased in METTL3-overexpression tumors (Figure 6D). The results from the knockdown and overexpression models led us to conclude that METTL3 played a critical role in promoting tumor growth *in vivo*.

Based on the mechanism we identified above, we proceeded to explore the clinical relevance of METTL3 and CDC25B in patient samples. Immunohistochemistry (IHC) staining of 39 clinical human cervical tumor tissues revealed that both METTL3 and CDC25B are moderately or highly expressed in most of the cervical cancer samples, whereas their expression is weak or not detectable in the superficial or intermediate squamous epithelial cell layer of the cervix (Fig. 6E). Interestingly, METTL3 and CDC25B are also moderately expressed in the normal parabasal/basal cell layer of the cervix squamous epithelium. The parabasal/basal cell layer is potentially generative mitotic activity may be seen (MIB-1 positivity) 32. This implies that METL3 and CDC25B are expressed in cells with high mitotic activity, whether it is tumor cells or normal cells. We further investigated the association between METTL3-CDC25B expression in cervical cancer samples. The immunohistochemical staining of METTL3 and CDC25B was categorized into either high or low expression, according to the IHC score, and the IHC scores of 39 samples are summarized in a heatmap (Figure 6F) (the final score was defined as the staining number score multiplied by the staining color score). Interestingly, high METTL3 expression tumors had high CDC25B (Figure 6G, P < 0.001) expression levels. Taken together, METTL3 and CDC25B expression are upregulated in human cervical cancer samples and the expression of CDC25B was related to that of METTL3.

## Discussion

Dysregulation of the cell cycle underlies the aberrant cell proliferation that characterizes cancer cells. Analysis of DNA replication-related molecules in cancer is now leading to the identification of novel biomarkers for cancer detection and is providing validated targets for cell cycle-targeted therapies. Here, we investigated the dynamic changes of gene mRNA levels in the cell cycle by RNA-seq analysis. We found that the mRNA and protein levels of METTL3 were significantly increased, which was associated with the highest m^6^A level in the M phase. Furthermore, METTL3 activation of the M-phase inducer CDC25B was dependent on YTHDF1-mediated m^6^A methylation, as determined by m^6^A sequencing and m^6^A MeRIP-qPCR assays. The expression of METTL3 is correlated with the expression of CDC25B in cervix cancer.

Recent studies have shown that depletion of YTHDF2 leads to the delay of mitotic entry due to the overaccumulation of negative regulators of the cell cycle, such as WEE1 33. Another study established that the demethylase FTO can phosphorylate and regulate *CCND1* and finally affect the G1 phase process 22. Le et al. proved that WTAP was involved in cell cycle arrest induced by TGFβ exposure, and SMAD2/3, JunB might be the targets of WTAP 34. Due to the short duration of cell division and the limitation of the synchronization method of previous studies, it remains unclear whether m^6^A is involved in the regulation of each cell cycle division. In the present study, we generated a cell cycle synchronization model using a thymine double blockade combined with a mitotic inhibitor to accurately capture the phases of cell cycle division. Stable expression of METTL3 in the G1, S and G2 phases was observed, which was consistent with previously reported experimental data and supported the reliability of this study 33. In the M phase samples collected with mitotic inhibitor specificity, we found an interesting phenomenon in which high expression of the methyltransferase METTL3 mediated an increase in methylation levels. Previous studies have shown that METTL3 promotes the proliferation of many types of cancer cells, such as liver, colon and gastric cancers 26, 35, 36. However, the regulation of METTL3 in the M phase has not been reported. In this study, we confirmed the regulatory effect of METTL3 on the M phase by using flow cytometry with a phosphorylated histone H3 antibody.

To explore the specific molecular mechanism by which METTL3 regulates the cell cycle, we performed methylation sequencing on samples in the G2 and M phases. The results showed that differentially methylated genes in the G2 and M phases are highly enriched in the cell cycle, cell proliferation and other related functions. In previous studies, the differentially expressed genes after METTL3 knockdown were significantly enriched in cell cycle-related functions, which also suggested the important role of METTL3 in the cell cycle 37. After further analysis of genes with differentially increased methylation levels in the M phase, we found that *CDC25B* is a candidate molecule for METTL3-mediated m^6^A modification during the M phase, and the methylation levels of its 3'UTR are increased during the M phase. As a key molecule involved in the regulation of the G2/M phase, CDC25B mainly affects G2-to-M phase switching by regulating the CDK1/CyclinB1 complex 5. Studies have shown that the mRNA level of *CDC25B* is constant during the cell cycle, while the protein level increases in the M phase, which is consistent with the results we observed 8. Follow-up experiments confirmed the regulatory effect of METTL3 on the protein expression level of CDC25B. It is known that the methylation recognition protein YTHDF1 can selectively recognize the methylation modification at the 3'UTR of the mRNA to regulate the translation level of the modified mRNA 38. This suggests that YTHDF1 is involved in the regulation of *CDC25B* via METTL3-mediated m^6^A modification. After YTHDF1 knockdown, the CDC25B protein level was downregulated concurrently with a decrease in the proportion of cells in the M phase. The above findings reveal new mechanisms by which METTL3-mediated m^6^A modification regulates the cell cycle.

Our *in vitro* and *in vivo* data revealed that regulation of the METTL3-m^6^A-CDC25B axis resulted in the adjustment to the proliferation of cancer cells. In light of the fact that the regulation of CDC25B expression by METTL3 in this study occurs at the translational level, we found METTL3 has no effect on the mRNA level of CDC25B. Therefore, previously RNAseq databases analysis could not find the role of METTL3 in regulating CDC25B. Furthermore, using an immunochemistry assay, we showed that the protein expression of METTL3 was related to CDC25B expression in cervical cancer specimens. These findings confirm that the METTL3-m^6^A-CDC25B axis is essential for the growth and maintenance of cancer cells and tumor progression.

In summary, this study demonstrates the role of METTL3 in phase transition during the cell cycle and reveals a novel METTL3/m^6^A/CDC25B pathway that is involved in cell cycle regulation. Our results not only broaden the understanding of m^6^A modification biology but also provide new targets for tumor diagnosis and treatment.

## Materials and Methods

### Human cervical tumor samples and cell culture

Samples were collected from patients who underwent surgery at the Shanghai First Maternity and Infant Hospital from 2014 to 2019, and the study was approved by the Institutional Ethics Committee of Shanghai First Maternity and Infant Hospital. The study was compliant with all the relevant ethical regulations regarding research involving human participants. Human cervical cancer HeLa cells and HEK-293T cells (#CCL-13&#FS-0156, ATCC, Rockville, MD, USA) were cultured in Dulbecco's modified Eagle's medium (DMEM) containing 10% fetal bovine serum (FBS) and 1% penicillin/streptomycin (#10378016, Gibco, Grand Island, NY, USA). All the cells were maintained in an atmosphere containing 5% CO_2_ at 37°C.

### Cell cycle synchronization

The cells were synchronized by the double-block method with thymidine and nocodazole as described in our previous study 25. Briefly, for the first block, HeLa cells were plated at 60% confluence and incubated in the presence of 2.5 mM thymidine for 18 hours. The cells were washed with PBS three times and released in the thymidine-free medium for 10 hours. For the second block, the cells were treated with 2.5 mM thymidine for 16 hours. The cells were considered synchronized at the G1/S boundary. Cells in the S phase and G1 phase were collected 4 and 15 hours later, respectively. Cells in the G2 phase were collected after being released and treated with nocodazole (100 ng/ml) for 8 h, and cells in the M phase were collected after being released and treated with nocodazole for 10 hours.

### Plasmids, siRNAs and transfection

Expression and purification of lentiviruses were performed as described in the manufacturer's protocol. For the generation of lentivirus encoding short hairpin RNA (shRNA) targeting METTL3, corresponding shRNA oligos were cloned into the pLKO.l vector (#8453, Addgene, Watertown, MA, USA). For lentiviral vectors expressing METTL3 and luciferase (control), the corresponding cDNA was cloned into the pKD-Puro-FLAG vector. To generate METTL3 stable knockdown and overexpression cells, HeLa cells were infected with lentivirus and selected with puromycin. Specific siRNAs targeting YTHDF1, and the control siRNA were purchased from IGE BIO (Guangzhou, China). Cell transfection was achieved by using Lipofectamine 2000 (#11668019, Invitrogen, Carlsbad, CA, USA) according to the manufacturer's protocol. All the shRNA and siRNA sequences are listed in Table S1.

### RT-Quantitative PCR

RT-qPCR was performed as described in our previous study 39. RNA was extracted according to the TRIzol RNA isolation protocol, cDNA was synthesized with a reverse transcriptase kit, and real-time PCR was performed with an SYBR Premix Ex Taq Kit (#RR820, Takara, Tokyo, Japan) in a CFX96 Real-Time PCR System (#185-5195, Bio-Rad, Hercules, CA, USA). Target gene expression levels were normalized against that of the 18S rRNA gene, and relative expression was calculated using the 2-ddCt method. All the primer sequences are listed in Table S2.

### Western blotting

Western blotting was performed as described in our previous study 40. Briefly, total protein lysates were prepared from cells with SDS buffer with proteinase. A BCA assay (P1002, Beyotime, Shanghai, China) was performed to quantify the protein concentrations. The obtained protein samples were resolved using 10% SDS-PAGE and transferred onto a PVDF membrane (#ISEQ00010, Millipore, Temecula, CA, USA). After being blocked with 5% fat-free milk, the membranes were cut into strips with molecular weight cut in half in-between the strips. Then, the strips were incubated overnight at 4°C with primary antibodies (the antibodies information was listed in Table S7). After incubation with secondary antibodies, the immunoreactive bands were visualized using an enhanced chemiluminescence reagent (WBKIS0100, Millipore, Temecula, CA, USA). The densities of the immunoreactive bands were determined by Quantity One 1-D Analysis Software, (RRID:SCR_014280).

### Measurement of the m^6^A levels

The m^6^A levels in the total RNA were detected using a commercial m^6^A RNA methylation quantification kit (#P9005, Epigentek, NY, USA). Briefly, 250 ng of total RNA was added to each well, and capture antibody solution and detection antibody solution were added according to the manufacturer's protocol. The absorbance of each well at a wavelength of 450 nm was measured to determine the m^6^A level, and then calculations were performed based on the standard curve.

### Flow cytometric cell cycle analysis

Briefly, cells were fixed in 70% alcohol overnight at -20°C and then incubated with 50 μg/ml propidium iodide (PI) and 100 μg/ml RNase A in the dark for 10 minutes. Cell cycle phase distribution was assessed using a FACScan flow cytometer (#342973, BD Biosciences, Franklin Lakes, NJ, USA). For the flow cytometry analysis of the M phase, fixed cells were treated with 0.25% Triton X-100 on ice for 15 minutes and then incubated with phospho-H3 antibody (#3465, CST, Danvers, MA, USA) (1:1500 in 1% BSA) for 3 hours at room temperature. The H3P-labeled cells were subsequently stained with PI and analyzed on a flow cytometer.

### CCK-8 assay

Cell proliferation was detected using a Cell Counting Kit-8 (CCK-8) assay according to the manufacturer's instructions. Briefly, cells were seeded into a 96-well plate at a density of 5000 cells/well. Cell growth was tested every day by adding 10 μl/well CCK-8 (#C0039, Beyotime, Shanghai, China), and the absorbance was read at 450 nm with a microplate reader (#ELX800, BioTek, Winooski, VT, USA). The average value was calculated from 5 replicates, and a growth curve was generated from the average values.

### Colony formation assay

A total of 400 cells were seeded in one well of a 6-well plate and maintained in the medium containing 10% FBS. After 2 weeks, the cells were fixed with 4% formaldehyde and stained with 0.1% crystal violet (#C0121, Beyotime, Shanghai, China). The number of colonies (defined as containing more than 50 cells) was determined under a light microscope, and each group included triplicate wells.

### RNA-sequencing (RNA-seq) and m^6^A sequencing (m^6^A-seq)

Based on the documented procedure, total RNA was extracted using TRIzol reagent (#15596026, Invitrogen, Carlsbad, CA, USA). The RNA-seq was performed using two biological replicate pools for each cell cycle phase, with each pool representing RNA from individual samples (n=3). The quality and quantity of the total RNA were detected using an RNA 6000 Nano LabChip Kit (#5067, Agilent, Santa Clara, CA, USA). A cDNA library was constructed and then sequenced with a Hiseq 4000 system (Illumina, CA, USA). Differentially expressed genes (DEGs) were identified using the DESeq2 package in R with thresholds pf a Benjamini-Hochberg adjusted p value <0.05 and a two fold change in expression. For m^6^A-seq, mRNA was purified using the Dynabeads mRNA Purification Kit (#61006, Thermo Fisher, Waltham, MA, USA). An anti-m^6^A antibody was used for the immunoprecipitation of m^6^A -modified mRNA. The input and immunoprecipitation samples were both used to construct the libraries. Then, the eluted RNA and MeRIPed RNA were analyzed by deep sequencing on an Illumina NovaseqTM 6000 platform at LC-BIO Ltd. (Hangzhou, China) following the vendor's recommended protocol. Reads were mapped to the human reference genome (GRC38) using R language, version 3.8.1. We filtered the differentially expressed peak genes based on a false discovery rate (FDR) of <0.05.

### MeRIP-qPCR

m^6^A modification of individual genes was assessed using a MeRIP-qPCR assay. Briefly, mRNA was purified from total RNA, and one-tenth of the RNA was saved as input control. After the RNA was fragmented, it was immunoprecipitated with an anti- m^6^A antibody or IgG coupled to Dynabeads (#1001, Thermo Fisher, Waltham, MA, USA). Further enrichment was calculated by qPCR, and the relative m^6^A enrichment in each sample was calculated by normalizing to the input.

### Tumorigenicity in nude mice

BALB/c-nu female mice (6 weeks old) (Hua Fu Kang, Beijing, China) were maintained in a specific pathogen-free animal room at the Jinan University Animal Center. Animal care and experimental procedures were approved by the Institutional Animal Care and Use Committees. Briefly, for the subcutaneously injected model, 5×10^6^ cells were injected into the flanks of the mice. The body weight and tumor volume were measured every two days. Tumor volume was assessed as (L×W^2^/2), where L and W represent the length and width, respectively. All the animals were killed at the end of the study, and their tumors were collected for further analysis.

### Histological and immunohistochemical (IHC) staining

Cervical cancer samples were cut into 4-μm-thick sections and placed on pathological slides for immunohistochemical staining. The sections were heated at 100°C in citrate buffer solution (pH=6.0) for 10 minutes to facilitate antigen retrieval. Then, the sections were incubated with rabbit antibodies against human METTL3 (EPR18810, Abcam, Cambridge, UK) and CDC25B (EPR3459(2), Abcam, Cambridge, UK) for 3 h followed by incubation with secondary antibodies (Dako REAL EnVision, USA). The immunoreacted cells were visualized using diaminobenzidine, and the nuclei were counterstained with hematoxylin. Phosphate-buffered saline (PBS) was substituted for the primary antibody as a negative control.

### IHC scores

To show the extent of immunostaining with indicated proteins anti-body, a semi-quantitative score was established. For this purpose, each sample was scored: (a) for the percentage of labeled cancer cells (0=absence; 1=less than 30%; 2=30-60%; and 3=more than 60%); and (b) and for the intensity of the immunostaining (0=no staining; 1=weak; 2=mild; and 3=strong staining). Multiplication of both scores allowed the final scoring of samples, ranging from 0 to 9. The expressions of indicated proteins were categorized as “low” for the score of “below 4.5” versus “high” for score of “above 4.5”. Analysis was performed by two independent pathologists.

### Statistical analysis

The data were graphed using GraphPad Prism 6 (RRID:SCR_002798) and analyzed to determine statistical significance by one-way analysis of variance (ANOVA) (for 3 or more comparisons) or one-tailed Student's t-test (for 2 group comparisons). The Kaplan-Meier method and log-rank tests were used to compare overall survival. p<0.05 was considered statistically significant. All the experiments were performed at least in triplicate. The data and error bars are presented as the mean ± standard deviation (SD) or standard error of the mean (S.E.M).

## Supplementary Material

Supplementary figure and tables.
