# Supplementary material for: METTL3 promotes cell cycle progression via m6A/YTHDF1-dependent regulation of CDC25B translation
Source: Int J Biol Sci. 2022 May 1;18(8):3223–36. doi: 10.7150/ijbs.70335 (PMC9134919; doi:10.7150/ijbs.70335)
Supplement: Supplementary file 1 — Supplementary figure and tables. [file ijbsv18p3223s1.pdf]

Supplementary Figure 1

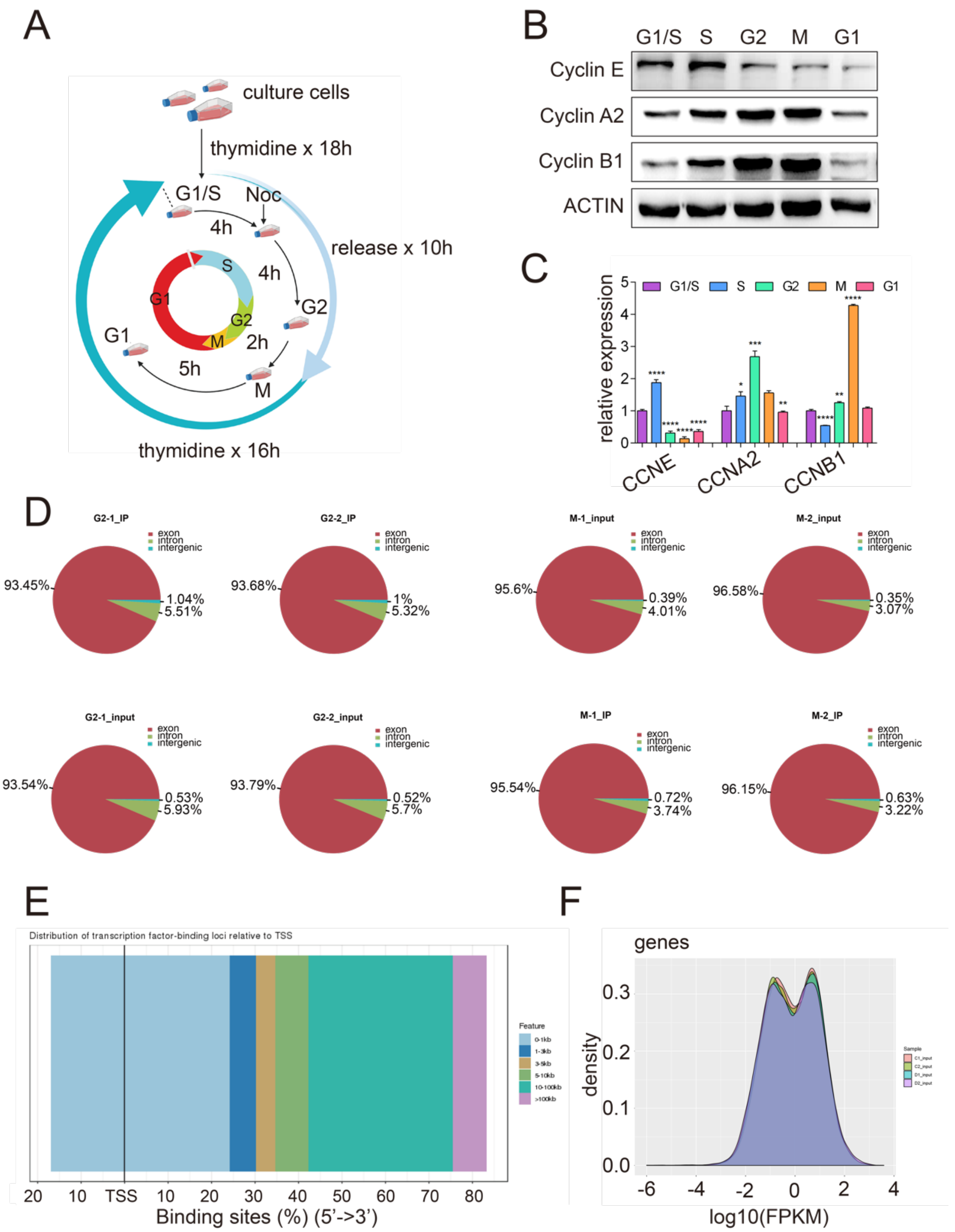

Figure S1. (A) Schematic of the double-block method with thymidine and nocodazole to synchronize the cell cycle. At the first block, Hela cells were treated with 2.5 mM thymidine for 18 hours and released in thymidine-free medium for 10 hours. After that, an additional block with 2.5 mM thymidine for 16 hours, cells in S phase and G1 phase were harvested respectively at 4 and 15 hours later. G2 and M phase cells

were further incubated with 100 ng/ml nocodazole for 8h and 10 hours after being released, respectively. Next, cells arrested in distinct phases were processed for further detection.

(B) Cell cycle synchronization efficiency of HeLa cells was examined by Western blotting of specific cell cycle markers (Cyclin A2, Cyclin B1 and Cyclin E). ACTIN served as a loading control.

(C) qPCR of *CCNA2*, *CCNB1* and *CCNE* was performed to detected cell cycle synchronization efficiency across differential cell cycle stages of HeLa cell. Relative mRNA expression levels were normalized by 18S rRNA. Data are presented as the mean ± SD of triplicate experiments. * $P < 0.05$, ** $P < 0.01$, *** $P < 0.001$, **** $P < 0.0001$ compared to the control group.

(D) Mapped region of each sample.

(E) Analysis of different m^6A peaks and Transcription Start Sites (TSS).

(F) Density of Different Expression Genes.

Table S1

Genes	Organism	Sequence (5'->3')
si YTHDF1#1	homosapien	GGACAGUCAAAUCAGAGUAdTdT
		UACUCUGAUUUGACUGUCCdTdT
si YTHDF1#2	homosapien	CCUACGGACAGCUCAGUAAdTdT
		UUACUGAGCUGUCCGUAGGdTdT
siNC		UUCUCCGAACGUGUCACGUdTdT
		ACGUGACACGUUCGGAGAAdTdT
shMETTL3#1	homosapien	AATTCCGTCAGTATCTTGGGCAAGTTCTCGAGAACTTGCCCAAGATACTGACGTTTTTT
		CTAGAAAAAACGTCAGTATCTTGGGCAAGTTCTCGAGAACTTGCCCAAGATACTGACGG
shMETTL3#2	homosapien	AATTCGCCAAGGAACAATCCATTGTTCTCGAGAACAATGGATTGTTCCTTGGCTTTTTT
		CTAGAAAAAAGCCAAGGAACAATCCATTGTTCTCGAGAACAATGGATTGTTCCTTGGCG
shNC	homosapien	CCTAAGGTTAAGTCGCCCTCGCTCGAGCGAGGGCGACTTAACCTTAGG
		CCTAAGGTTAAGTCGCCCTCGCTCGAGCGAGGGCGACTTAACCTTAGG

Table S2

Genes	Primers	Organism	Sequence (5'->3')
GAPDH	Forward	homosapien	ACAACTTTGGTATCGTGGAAGG
GAPDH	Reverse	homosapien	GCCATCACGCCACAGTTTC
CCNA2	Forward	homosapien	GAGGATATTCACACATACCTTAGGG
CCNA2	Reverse	homosapien	TCCACGAGGATAGCTCTCATAC
CCNB1	Forward	homosapien	CTTTCGCCTGAGCCTATTTTG
CCNB1	Reverse	homosapien	ACATCAGAGAAAGCCTGACAC
CCNE	Forward	homosapien	GCCAAAATCGACAGGACGG
CCNE	Reverse	homosapien	ATCTTCTTTGTCAGGTGTGGG
METTL3	Forward	homosapien	AGAGCCTTCTGAACCAACAG
METTL3	Reverse	homosapien	CAATGGATTGTTCCTTGGCTG
METTL14	Forward	homosapien	GAGCTGAGAGTGCGGATAGC
METTL14	Reverse	homosapien	GCAGATGTATCATAGGAAGCCC
FTO	Forward	homosapien	ACTTGGCTCCCTTATCTGACC
FTO	Reverse	homosapien	TGTGCAGTGTGAGAAAGGCTT
ALKBH5	Forward	homosapien	CATACGGCCTCAGGACATC
ALKBH5	Reverse	homosapien	TCAGGGACTTTGTTTCCAACC
CDC25B	Forward	homosapien	CAGGTCTCTGCATGGATTCC
CDC25B	Reverse	homosapien	TGCTCGTTTCGAATGATCCG
YTHDF1	Forward	homosapien	GACTTTGAGCCCTACCTTACTG
YTHDF1	Reverse	homosapien	CAATGGACGGCGGGTAATAG

Table S6

Term	Count	%	PValue	Genes	List Total	Pop Hits	Pop Total	Fold Enrichment	FDR
GO:0000398~mRNA splicing, via spliceosome	39	0.0187	5.65E-07	RALY, POLR2F, POLR2L, POLR2J, TRA2B, SYNCRIP, SART3, BUD31, CACTIN, XAB2, SART1, HNRNPM, DHX38, METTL3, HNRNPF, SNRNP70, DHX35, DDX41, DDX39A, SNRPA1, PRPF31, CSTF3, SPEN, SF3A2, SF3A3, UBL5, PPIE, PCF11, EIF4A3, SRSF5, RBPMS, PAPOLA, AQR, GTF2F1, RSRC1, SNRNP25, PDCD7, CPSF1, SNRPG	1217	222	16792	2.423949011	0.001
GO:0006260~DNA replication	23	0.011	0.0018118	POLL, ING5, ACHE, RECQL5, DTL, POLG, LIG1, TREX1, IGF1, NFIX, RMI2, RBBP7, MCM5, RPA3, CDT1, SET, ORC4, SUPT16H, CHTF18, POLG2, E4F1, RTEL1-TNFRSF6B, ATRIP	1217	155	16792	2.047424921	3.2978
GO:0006281~DNA repair	28	0.0134	0.0116046	KIF22, RAD51C, STUB1, RPA3, EPC2, UBE2D3, CHRNA4, POLG2, RTEL1-TNFRSF6B, INO80C, INO80B, POLL, RECQL5, NUDT1, LIG1, TRIM28, SMG1, TREX1, RAD51, DOT1L, PNKP, UHRF1, SUPT16H, RAD18, RUVBL2, OGG1, GADD45A, USP45	1217	235	16792	1.644000769	19.414
GO:0051436~negative regulation of ubiquitin-protein ligase activity involved in mitotic cell cycle	12	0.0057	0.0126872	ANAPC2, PSMD14, PSMC5, PSMD12, PSME1, PSMB2, PSMD2, UBC, PSMD4, ANAPC11, CDC26, UBE2E1	1217	71	16792	2.332033284	21.031

Table S6 Continued

GO:0045931~positive regulation of mitotic cell cycle	6	0.0029	0.0481613	SHB, EIF4EBP1, PKN2, PAFAH1B1, CDC25B, TERF1	1217	28	16792	2.956685057	59.859
GO:0006977~DNA damage response, signal transduction by p53 class mediator resulting in cell cycle arrest	9	0.0043	0.0776215	CNOT9, BAX, CNOT10, CNOT3, UBC, ARID3A, RBM38, PIDD1, GADD45A	1217	62	16792	2.002915684	77.556
GO:0008283~cell proliferation	36	0.0172	0.0555665	ACHE, HRAS, PPARD, GNAI2, ERBB2, BCAR1, SIPA1, BOP1, SCRIB, PRMT5, CKLF, ILK, E4F1, DDX41, BHLHE41, NUDC, MATK, EGFR, IRS2, CRIP1, PDXK, IGF1, RBBP7, TACC2, UHRF1, IGSF8, PRDM4, LAMA5, SIX1, H3F3A, TCF19, BIN1, EMP2, EMP1, IGFBP4, MAP3K11	1217	366	16792	1.357166911	65.257
GO:0051301~cell division	35	0.0167	0.0493404	MAD1L1, CLTA, LZTS2, HAUS5, GNAI3, GNAI2, BCAR1, NEDD9, CDC34, ANAPC11, CDC37, SPC25, FIGN, CCDC124, KLHL21, E4F1, NSUN2, KMT5A, NUDC, TERF1, CSNK1A1, PARD6A, ANAPC2, RECQL5, LIG1, PSRC1, PKN2, KIF18B, CDC26, PPP1CC, MARK4, MCM5, CDC25B, CCND1, CENPT	1217	350	16792	1.37978636	60.769

Table S6 Continued

GO:0006977~DNA damage response, signal transduction by p53 class mediator resulting in cell cycle arrest	9	0.0043	0.0776215	CNOT9, BAX, CNOT10, CNOT3, UBC, ARID3A, RBM38, PIDD1, GADD45A	1217	62	16792	2.002915684	77.556
GO:0006412~translation	26	0.0124	0.0701683	MRPL2, EGFR, MRPL4, SLC25A6, MRPS12, RPL26, RPS6KB2, RPL36, RPL37, RPL38, UCP3, RPL31, UCP2, RPS14, SLC25A10, MRPL17, SLC25A22, RPL26L1, SLC25A29, SLC25A37, MRPL55, SLC25A44, SLC25A39, EEFSEC, MRPL43, RMND1	1217	253	16792	1.417962267	73.955

Table S7

ANTIBODIES	SOURCE	CAT No
GAPDH Polyclonal antibody	Proteintech Group	10494-1-AP
METTL3 Polyclonal antibody	Proteintech Group	15073-1-AP
FTO Polyclonal antibody	Proteintech Group	27226-1-AP
ALKBH5 Polyclonal antibody	Proteintech Group	16837-1-AP
Anti-cyclin E Antibody (E-4)	Santa Cruz Biotechnology	sc-377100
Cyclin A2 Polyclonal antibody	Proteintech Group	18202-1-AP
Cyclin B1 Polyclonal antibody	Proteintech Group	55004-1-AP
Beta Actin Polyclonal antibody	Proteintech Group	20536-1-AP
CDC25B Polyclonal antibody	Proteintech Group	10644-1-AP
YTHDF1 Polyclonal antibody	Proteintech Group	17479-1-AP
Anti-METTL14 antibody produced in rabbit	MilliporeSigma	HPA038002
WTAP Monoclonal antibody (4A10G9)	Proteintech Group	60188-1-Ig
